# Eph Receptors Are Involved in the Activity-Dependent Synaptic Wiring in the Mouse Cerebellar Cortex

**DOI:** 10.1371/journal.pone.0019160

**Published:** 2011-04-29

**Authors:** Roberta Cesa, Federica Premoselli, Annamaria Renna, Iryna M. Ethell, Elena B. Pasquale, Piergiorgio Strata

**Affiliations:** 1 Department of Neuroscience, University of Turin, Turin, Italy; 2 National Neuroscience Institute-Italy at Turin University, Turin, Italy; 3 Division of Biomedical Sciences, University of California Riverside, Riverside, California, United States of America; 4 Sanford-Burnham Medical Research Institute, La Jolla, California, United States of America; University of Cincinnatti, United States of America

## Abstract

Eph receptor tyrosine kinases are involved in many cellular processes. In the developing brain, they act as migratory and cell adhesive cues while in the adult brain they regulate dendritic spine plasticity. Here we show a new role for Eph receptor signalling in the cerebellar cortex. Cerebellar Purkinje cells are innervated by two different excitatory inputs. The climbing fibres contact the proximal dendritic domain of Purkinje cells, where synapse and spine density is low; the parallel fibres contact the distal dendritic domain, where synapse and spine density is high. Interestingly, Purkinje cells have the intrinsic ability to generate a high number of spines over their entire dendritic arborisations, which can be innervated by the parallel fibres. However, the climbing fibre input continuously exerts an activity-dependent repression on parallel fibre synapses, thus confining them to the distal Purkinje cell dendritic domain. Such repression persists after Eph receptor activation, but is overridden by Eph receptor inhibition with EphA4/Fc in neonatal cultured cerebellar slices as well as mature acute cerebellar slices, following *in vivo* infusion of the EphA4/Fc inhibitor and in EphB receptor-deficient mice. When electrical activity is blocked *in vivo* by tetrodotoxin leading to a high spine density in Purkinje cell proximal dendrites, stimulation of Eph receptor activation recapitulates the spine repressive effects of climbing fibres. These results suggest that Eph receptor signalling mediates the repression of spine proliferation induced by climbing fibre activity in Purkinje cell proximal dendrites. Such repression is necessary to maintain the correct architecture of the cerebellar cortex.

## Introduction

Ephrin ligands and their receptors, the Eph receptor (Eph) tyrosine kinases, are cell surface molecules that mediate communication between cells. They are involved in multiple cellular processes in different tissues, where they regulate cell shape and position. Ephrins and Eph receptors are especially highly expressed in the embryonic nervous system, where their signalling is important for proper axonal pathfinding and to establish topographic projections [1]–[3]. Furthermore, they maintain a remarkable presence in the adult brain, especially in regions characterized by a high degree of structural plasticity in adulthood, such as the hippocampus, olfactory bulb and cerebellum [2], [4]–[6].

Recent studies in the hippocampus and cerebral cortex show that Eph receptors and ephrins are important in the regulation of synaptic morphology and plasticity. For example, in *EphB1/EphB2/EphB3*-deficient mice dendritic spines (the small protrusions bearing postsynaptic structures) have reduced density and size [3], [7], [8] while activation of EphB receptors by ephrins induces rapid dendritic spine formation [7], [9]. EphA receptors have also been implicated in the regulation of spine development and stability [7], [8], [10]–[12]. Activation of EphA4 in acute slices from adult mouse hippocampus by administration of a soluble form of its ligand ephrinA3 triggered the retraction and pruning of spines within a time span of only 45 minutes while decreased EphA4 signalling induced long and irregularly shaped spines [10]. Interestingly, Eph receptors can also be found in presynaptic terminals [3], [13] and their cell surface expression can be regulated by synaptic activity [14].

The cerebellum is endowed with a high degree of structural plasticity even in adulthood and it can undergo remarkable activity-dependent architectural changes [15], [16].

The Purkinje cells of the cerebellar cortex are innervated by two different excitatory inputs: the parallel fibres and the climbing fibres, which contact two separate dendritic domains, different for size and spine density.

The proximal dendritic domain has a diameter above 2 µm with a very low number of spines and extends up to near the surface of the cortex. In contrast, the distal dendritic domain is made by a high number of branchlets with the highest spine density in the brain (spiny branchlets) [17] abutting from the proximal domain at all cortical levels.

Each climbing fibre, representing the terminal arborisation of an inferior olivary axon, has a low number varicosities [18] and each varicosity makes excitatory contacts on a cluster of 2–6 spines emerging from the proximal domain of a single Purkinje cell. In contrast, many parallel fibres (which are the axons of cerebellar granule cells) form synapses with the spines of the distal dendritic compartment of a Purkinje cell.

In mutant mice in which parallel fibre to Purkinje cell synapses are impaired, there is an extension of the climbing fibre territory into the distal domain [19]. In contrast, in response to climbing fibre degeneration after subtotal lesion of inferior olivary neurons by means of intraperitoneal injections of 3-acetylpyridine [20], [21], an intense spine proliferation occurs only in the proximal Purkinje cell dendritic domain. The new spines appear on the dendritic surface between the varicosities of climbing fibres and are innervated by parallel fibres [20]–[23]. A similar hyperspiny transformation in the proximal Purkinje cell dendritic domain occurs following blockade of electrical activity by *in vivo* intraparenchymal administration of tetrodotoxin [24]–[26]. When Purkinje cells are reinnervated by the climbing fibres, either upon removal of tetrodotoxin or by the collateral sprouts that form following subtotal 3-acetylpyridine lesion, a normal spine distribution pattern is restored. Thus, parallel fibre and climbing fibre inputs are in a continuous state of competition. These findings led to the hypotheses that: i) an activity-independent, intrinsic mechanism, promotes spine growth over the whole dendritic territory of Purkinje cells [22], [27], [28] and ii) an activity-dependent spine-pruning action is exerted by the climbing fibres near their synapses in the proximal dendrites [19], [24], [25], [29].

A number of Eph receptors and ephrins of both the A and B classes are expressed in complex patterns in cerebellar neurons, including Purkinje cells, both during development and in adulthood [4], [30]–[37]. However, their effects on spinogenesis have not been addressed in the cerebellum.

By using both gain-of-function and loss-of-function strategies to selectively activate or inhibit EphA and EphB receptors both *in vivo* and *in vitro*, we uncovered the involvement of Eph receptor signalling in the structural plasticity of Purkinje cell spines in the adult cerebellum. Eph receptor signalling appears to be responsible for the activity-dependent spine repression of spinogenesis that operates in the proximal dendritic domain of the Purkinje cell.

## Methods

### Animals

The study was performed on neonatal (body weight, 1–5 g; age, 10 days) and adult mice (body weight, 30–35 g; age, 40–50 days). L7-EGFP BAC transgenic mice (Gensat Project, Rockefeller University, NY), in which the reporter gene is selectively expressed by the Purkinje cells, were used for the *in vitro* experiments. CD1 mice (Harlan Italy, Milano, Italy) were used for the *in vivo* experiments. All surgical procedures were performed under general anaesthesia by a mixture of ketamine (100 mg/kg Ketavet; Gellini Farmaceutici, Latina, Italy) and xylazine (5 mg/kg Rompum; Bayer, Leverkusen, Germany). Double *EphB1^−/−^*, *EphB3^−/−^* and triple *EphB1^−/−^*, *EphB2^+/−^*, *EphB3^−/−^* deficient mice were previously generated [7] and housed in the laboratory of Dr Iryna Ethell. The experimental plan was designed in accordance with the Council Directive of November 24, 1986 (86/609/EEC) of the European Community, the National Institutes of Health guidelines, and the Italian law for care and use of experimental animals (DL116/92) and was approved by the Italian Ministry of Health and the Bioethical Committee of the University of Turin.

### Ephrin receptor and ligand chimeras

Chimeras were obtained from R & D Systems (Minneapolis, Minnesota) and resuspended in sterile water. For *in vitro* experiments, chimeras were used at a concentration of 5.0 µg/ml of culture medium for organotypic cultures or of artificial cerebro-spinal fluid (ACSF), for acute slices preparations. Chimeras are Fc fusion proteins in which the Fc portion of human immunoglobulin G1 is fused to the extracellular domain of mouse ephrin ligands or receptors via a polypeptide linker. For *in vivo* infusion, 7 µg/day of chimeras in saline were delivered by osmotic minipumps. Like the membrane-bound ligands, Fc-clustered ephrins are able to activate the receptors inducing their autophosphorylation. On the contrary, Fc-bound Eph receptors compete with the endogenous Eph receptors for binding to membrane-bound endogenous ligands, thus inhibiting the endogenous ephrin/EphB signalling.

### Organotypic cultures

Anaesthetised P10 L7-GFP mouse pups were transcardially perfused with 5 ml of saline (0.9% NaCl) with 0.6% glucose, to remove blood. The brains were rapidly removed and placed into the same chilled buffer and the meninges were removed carefully. Cerebellar parasagittal slices, 250 µm thick, were cut using a McIllwain tissue chopper and cultured on the membrane of 30 mm Millipore (Millicell, Millipore, Bedford, MA, USA; pore size 0.4 µm) culture dishes placed in 35 mm culture dishes containing 1 ml culture medium composed of 50% basal medium with Earle's salts (In Vitrogen, Life Technologies Corporation, Carlsbad, California), 2.5% HBSS (In Vitrogen), 25% horse serum (In Vitrogen), 22.5% water, 1 mM l-glutamine (In Vitrogen), 200 U/ml penicillin/streptomycin (In Vitrogen) and 5 mg/ml glucose. The slices were kept at 37°C in a humidified atmosphere with 5% CO_2_. One day after the preparation, the culture medium was replaced with a medium containing chimeras except for the control slices in which the same amount of sterile water was added. After 2 or 24 hours the culture medium was replaced with 4% paraformaldehyde (PAF) in 0.12 M phosphate buffer (PB) pH 7.4 and the organotypic slices were fixed overnight at 4°C. The slices were detached carefully from the Millicell membrane before being processed for immunohystochemistry. At least 8 slices for each experimental group were used for quantitative analysis.

### Acute slices

Anaesthetised adult mice were decapitated and the vermis of the cerebellum was removed and quickly cooled by dipping in ice-cold ACSF (125 mM NaCl, 2.5 mM KCl, 1.25 mM NaH_2_PO_4_, 1 mM MgCl_2_, 2 mM CaCl_2_, 26 mM NaHCO_3_, 20 mM glucose) saturated with 95% O_2_ and 5% CO_2_. Parasagittal slices, 200 µm thick were cut in a vibratome (Vibroslice 752; Campden Instruments, Sileby, UK) and kept alive for at least 4 hours after preparation by using a protocol usually adopted for electrophysiological recordings that allows to analyse the cells several hours after cutting the cerebellum [38], [39]. In particular, they were left to recover 1 hr in ACFS at 25°C. Then, slices were incubated at room temperature in oxygenated ACSF with either soluble ephrinA2/Fc or ephrinB1/Fc for stimulation experiments, with EphA4/Fc to inactivate both EphA and EphB pathways, or with ACSF only as control. After 1 or 3 hours of incubation, the slices were fixed with 4% PAF in 0.12 M PB buffer, pH 7.4, for 3 hours before being processed for immunohystochemistry. At least 3 slices for each experimental group were used for quantitative analysis.

### *In vivo* chemicals administration

To interfere with Eph/ephrin pathways *in vivo*, we chronically infused for 6 days the chimeras into the dorsal vermal cortex by means of an osmotic minipump (Alzet 2002; Alza, Cupertino, CA), with the brain cannula inserted into the sixth lobule of the vermis. Vehicle mice were infused only with physiological solution, whereas control mice with recombinant human IgG_1_ Fc only. Tetrodotoxin (1 µM in saline; Tocris Cookson, Bristol, UK) was infused for 6 days alone or with the chimeras. At the end of the survival period, under general anaesthesia, mice were transcardially perfused with 4% PAF in 0.12 M PB buffer, pH 7.4. The cerebella were dissected, kept in the fixative overnight at 4°C, cryoprotected, cut in several series of 30 µm-thick sagittal sections using a cryostat and processed for immunohystochemistry. At least 3 mice for each experimental group were used for quantitative analysis.

### *EphB* deficient mice cerebella

Adult double *EphB1^−/−^*, *EphB3^−/−^* deficient mice (2 animals) and triple *EphB1^−/−^*, *EphB2^+/−^*, *EphB3^−/−^* deficient mice (2 animals) were asphyxiated with carbon dioxide inhalation and immediately perfused with PBS followed by 4% PAF. The cerebella were extracted and post-fixed in 4% PAF overnight at 4°C. After the cryoprotection, series of 30 µm-thick sagittal sections were prepared from the cerebella using a cryostat and processed for immunohystochemistry as described below.

### Histological procedures

We used a mouse monoclonal calbindin D-28K antibody (1∶2000; Swant, Bellinzona, Switzerland) to label Purkinje cells. In order to visualize parallel and climbing fibre terminals we used an antibody raised in rabbit against the vesicular glutamate transporter 1 (VGlut1; 1∶1000) selectively expressed by parallel fibres and one against the vesicular glutamate transporter 2 (VGlut2; 1∶500) to visualize climbing fibre varicosities [40]. Organotypic and acute slices were incubated for 2 nights at 4°C with the primary antibodies, whereas the minipump-treated mice sections for one night. The following day, slices and sections were treated for 1 h with the following secondary antibodies: horse anti-mouse coupled to fluorescein isothiocyanate (1∶200; Millipore, Bedford, MA) and biotinylated goat anti-rabbit (1∶200; Vector Laboratories, Burlingame, CA) coupled with Texas Red avidin (1∶200; Vector Laboratories). The reacted slices and sections were mounted on slides and coverslipped. Fluorescent immunostained sections were used to obtain images with a Fluoview confocal laser-scanning microscope (Olympus Optical). The diffusion of the chimeras in the parenchyma by means of minipumps was evaluated with a biotinilated donkey anti-human IgG antibody (Jackson ImmunoResearch, USA; 1∶200), which binds the IgG portion of the chimeras, and was visualized by diaminobenzidine immunohystochemistry. In particular, sections were incubated overnight at 4°C with the primary antibody. Subsequently they were exposed for 1 h at RT to the avidin-biotin-peroxidase complex (Vectastain, ABCElite Kit; Vector Laboratories, Burlingame, Inc., CA, USA). Histochemical detection of the peroxidase activity was carried out by incubation in 0.1 M TrisHCl buffer, pH 7.4, containing 3% 3.3-diaminobenzidine (MP Biomedicals, Illkirch, France) and 0.015% hydrogen peroxide. After rinsing, the sections were mounted onto chrome alum gelatinised slides, air dried, dehydrated, coverslipped and observed under an optical microscope.

### Confocal and optical imaging

Confocal imaging and spine density analysis were performed as previously described [39]. For each slice of *in vitro* experiments or cerebellum considered for the quantitative analysis, we randomly acquired five images of the molecular layer with at least one segment of Purkinje cell proximal dendrite. Proximal and distal dendrites were identified according to the two different calibres and only those having a diameter above and below 2 µm, respectively, were retained for quantitative analysis [24]. We used a 100× oil-immersion lens and an additional electronic zoom factor of 1.5× to clearly resolve dendritic spines. We collected a variable number of optical section images in the *z*-dimension (*z*-spacing, 0.5 µm) ensuring that segments of dendrites, spanning multiple confocal planes, were fully captured. The same images were used to measure the spine density in proximal and distal dendritic segments, whose lengths ranged between 5 and 50 µm and between 2 and 12 µm respectively. The figures provide images derived from the projection of a series of different optical sections spanning multiple confocal planes, in order to visualize the entire dendrite, but the spine density evaluation was calculated by collecting only the spines emerging from the proximal and distal dendrite in the central image of the series. To this aim, care was taken to select only completely isolated dendritic segments. In detail (see Figure S1 for an example of spine density evaluation on distal dendrites), each spine emerging from the dendritic segment in the central optical section was followed until it disappeared downstream and upstream in the image series in order not to include spines emerging from surrounding dendrites. Other spines near the dendrite have not been included in the counts, although they were in the central optical section and belonging to the same dendrite, as revealed by the examination of adjacent sections. Therefore, the quantitative spine evaluation was underestimated if compared to the total number of spines. To definitely evaluate their total number, an electron microscopy analyses performed on serial ultrathin section to reconstruct the entire dendritic surface would be required. However, the method we used is suitable to detect and compare relative spine density differences among different experimental groups and not to provide absolute spine numbers. The lengths of sampled proximal and distal dendrites were measured by means of ImageJ (Wayne Rasband, National Institutes of Health, USA) software. The spine density was expressed as the number of spines per micrometer of dendrite membrane length (*n* in brackets means the number of sampled dendrite membrane lengths). For the evaluation of the climbing fibre terminal arbour, the same optical section images were projected into a single one: all labelled varicosities distributed along the proximal dendrites were counted. The lengths of sampled proximal dendrites and varicosities were measured by means of ImageJ software to calculate the number of labelled varicosities per micrometer of dendrite membrane length, major and minor axis, area and perimeter of each varicosity (*n* in brackets means the number of sampled dendrite segments), according to a method already described that was also confirmed by an ultrastructural analysis [39].

### Statistical analysis

Comparisons between two independent groups were made using *t* tests with a two-tailed confidence level of *P*<0.05. Multiple comparisons were performed with one-way ANOVA. If the ANOVA revealed a significant difference (*P*<0.05) between groups, Student-Newman-Keuls post hoc test was used for pairwise multiple comparisons. All data represent mean ±s.e.m. The number of samples, *n*, is indicated in brackets in the tables. Statistical tests were run on SigmaStat 2.0 (Jandel Scientific Software).

## Results

To examine the involvement of Eph receptors in the activity-dependent remodelling of the cerebellar cortex, we activated or inhibited these receptors in cultured organotypic slices of neonatal cerebellum, in acute slices of mature cerebellum and in *in vivo* models of wild type mice by administering chimeras that have been extensively used to study Eph/ephrin signalling both *in vitro* and *in vivo* [9], [10], [41]. We used ephrinA2/Fc and ephrinB1/Fc to activate class A and B Eph receptors, respectively. Furthermore, because EphA4 is known to bind ephrins of both A and B classes [3], [42], we used EphA4/Fc to inhibit all Eph receptor pathways. In addition, we analysed the cerebellum of *EphB*-deficient mice.

### Activation of class A and B Eph receptors in neonatal cerebellar slice cultures does not affect dendritic spine density in Purkinje cells

**Figure 1:**
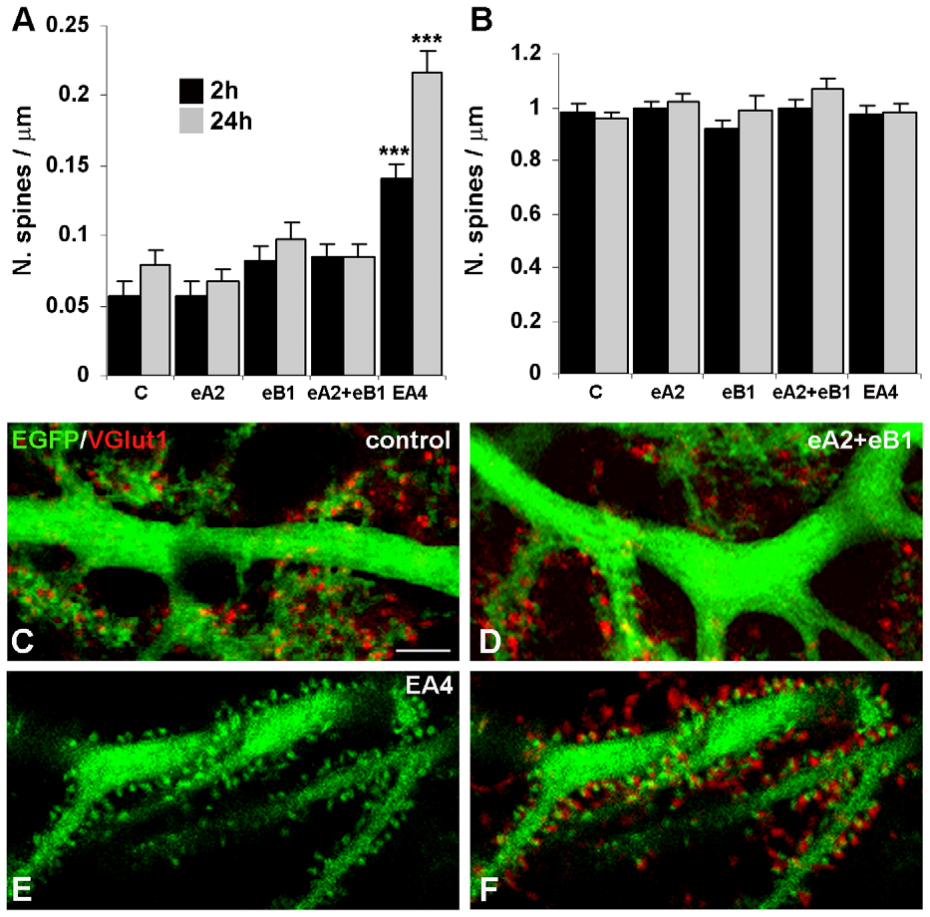
Purkinje cell morphological modifications in P10 organotypic cultures treated with ephrin ligand/and receptor/Fc chimeras. ***A, B,*** The histograms show the mean density of spines emerging from the proximal (***A***) and distal (***B***) dendritic domain after 2 and 24 hours of treatment with various ephrin and Eph receptor Fc chimeras. Only EphA4/Fc (EA4) treatment significantly increased spine density in the proximal compartment relative to the control treatment (C). ***P<0.001, Student's t-test. Error bars indicate s.e.m.. eA2, ephrinA2/Fc; eB1, ephrinB1/Fc. ***C, D, E, F,*** Confocal images of EGFP-expressing proximal dendrites (green) after 2 hours of treatment. The Purkinje cell proximal dendritic domain is smooth under control conditions (***c***) and after ephrinA2/Fc plus ephrinB1/Fc administration (***D***). The parallel fibres, selectively labelled with the antibody for VGlut1, contact spines in the distal domain (red in ***C*** and ***D***). Following inactivation of EphA and EphB receptor pathways with EphA4/Fc, proximal dendrites are covered by spines (***E***) that are contacted by VGlut1-positive parallel fibres (red in ***F***). Scale bar, 10 µm.

In a first group of experiments, we altered Eph/ephrin signalling during development in organotypic slices from the P10 cerebellum of L7-EGFP mice, which express enhanced green fluorescent protein (EGFP) in Purkinje cells. At P10–P11 Purkinje cell dendrites have already developed a proximal compartment with very low spine density and a distal compartment that is densely covered by spines that are contacted by parallel fibres [19]. After one day in culture, we administered ephrinA2/Fc, ephrinB1/Fc or a mix of both into the culture medium for 2 or 24 hours and then fixed the slices and evaluated spine density by confocal microscopy. As shown in Table 1A, average spine density values were not significantly different in treated and control groups in the proximal dendritic domain at 2 (F_(3,269)_ = 2.59, *P*>0.05, one-way ANOVA) and 24 hours (F_(3,285)_ = 1.42, *P*>0.2, one-way ANOVA) or in the distal domain at 2 (F_(3,195)_ = 1.67, *P*>0.17, one-way ANOVA) and 24 hours (F_(3,190)_ = 1.69, *P*>0.17, one-way ANOVA) (Fig. 1A and 1B). Figures 1C and 1D show representative examples of a control slice and a slice treated with both ephrinA2/Fc and ephrinB1/Fc for 2 hours.

**Table 1:** Spine density on the proximal and distal dendritic domains of the Purkinje cells in different experimental approaches.

		Spine density in the proximal dendritic domain		Spine density in the distal dendritic domain	
**A.**	**Neonatal organotypic slice cultures**				
		2 hours	24 hours	2 hours	24 hours
	Control	0.057±0.011 (n = 73)	0.079±0.011 (n = 69)	0.983±0.030 (n = 49)	0.954±0.027 (n = 49)
	eA2	0.057±0.010 (n = 71)	0.067±0.009 (n = 75)	1.000±0.023 (n = 49)	1.020±0.034 (n = 49)
	eB1	0.083±0.010 (n = 61)	0.097±0.012 (n = 70)	0.918±0.028 (n = 51)	0.992±0.051 (n = 47)
	eA2+eB1	0.086±0.009 (n = 68)	0.085±0.010 (n = 75)	0.993±0.035 (n = 50)	1.070±0.036 (n = 49)
	EA4	0.140±0.011 (n = 95)***	0.217±0.015 (n = 96)***	0.975±0.029 (n = 52)	0.977±0.034 (n = 49)
**B.**	**Acute mature slices**				
		1 hour	3 hours	1 hour	3 hours
	Control	0.059±0.014 (n = 18)	0.067±0.019 (n = 19)	1.023±0.025 (n = 25)	1.093±0.033 (n = 25)
	eA2	0.060±0.015 (n = 20)	0.099±0.017 (n = 33)	1.088±0.034 (n = 25)	1.082±0.034 (n = 25)
	eB1	0.058±0.014 (n = 22)	0.111±0.023 (n = 32)	1.078±0.036 (n = 25)	1.057±0.034 (n = 25)
	EA4	0.219±0.027 (n = 24)***	0.199±0.035 (n = 29)**	1.050±0.027 (n = 25)	1.032±0.026 (n = 25)
**C.**	**6 days of infusion of chimeras**				
	Vehicle	0.083±0.011 (n = 106)		0.937±0.034 (n = 59)	
	Fc	0.093±0.012 (n = 96)		0.949±0.020 (n = 60)	
	eA2	0.092±0.011 (n = 103)		0.934±0.028 (n = 59)	
	eB1	0.093±0.011 (n = 102)		0.966±0.032 (n = 60)	
	eA2+eB1	0.109±0.010 (n = 109)		0.994±0.023 (n = 60)	
	EA4	0.321±0.030 (n = 87)*		0.996±0.022 (n = 59)	
**D.**	**EphB deficient mice**				
	Wild type	0.088±0.024 (n = 41)		0.968±0.023 (n = 61)	
	EphB1−/−EphB2+/+EphB3−/−	0.071±0.014 (n = 41)		0.979±0.025 (n = 27)	
	EphB1−/−EphB2+/−EphB3−/−	0.149±0.017 (n = 40)*		0.962±0.019 (n = 60)	
**E.**	**6 days of infusion of TTX only or with chimeras**				
	TTX	0.437±0.018 (n = 84)		0.973±0.017 (n = 109)	
	Fc+TTX	0.447±0.028 (n = 100)		0.964±0.017 (n = 59)	
	eA2+TTX	0.228±0.022 (n = 66)*		0.930±0.015 (n = 58)	
	eB1+TTX	0.240±0.021 (n = 65)*		0.989±0.019 (n = 73)	
	eA2+eB1+TTX	0.271±0.032 (n = 62)*		1.008±0.016 (n = 63)	

Mean values of spine densities expressed as number of spines per µm of dendritic length, ±s.e.m.. In A and B, the asterisks indicate the significance between the group treated with EphA4/Fc and the control, performed with the Student's t-test. In C, the asterisk indicates the significance between the group treated with EphA4/Fc, the control and the negative control Fc, performed with one way ANOVA followed by the post hoc Student-Newman-Keuls method. In D and E, pairwise multiple comparisons between groups were performed with one way ANOVA followed by the post hoc Student-Newman-Keuls method. The asterisk in D indicate a statistically significant difference between the latter group and the others, whereas in E the post hoc method indicates a significant difference between the groups treated with ligand chimeras in addition to TTX and those treated with TTX and Fc+TTX, but not between each other. Abbreviations used: eA2, ephrinA2/Fc; eB1, ephrinB1/Fc; EA4, EphA4/Fc; Fc, portion of recombinant human IgG1 (negative control); TTX, tetrodotoxin.

### Inhibition of Eph receptors in neonatal cerebellar slice cultures increases spine density in the proximal, but not in the distal domain of Purkinje cell dendrites

To inhibit both class A and B Eph receptors, we treated slices with EphA4/Fc chimeras. The treatment resulted in the formation of new spines in the proximal dendritic territory, which were contacted by parallel fibres (Table 1A and Fig. 1A,E,F). Both at 2 and 24 hours of treatment the mean spine density values in the treated slices were significantly higher relative to controls fixed at the same experimental time periods (*P*<0.001 in both cases, Student's t-test). In contrast, the mean spine density values in the distal dendritic domain of treated and control slices were not significantly different (*P*>0.6 for both 2 and 24 hours, Student's t-test) (Table 1A, Fig. 1B). Thus, pharmacological inhibition of Eph receptor signalling in organotypic cerebellar slices can induce spine formation in the Purkinje cell proximal dendritic domain but not in the distal domain. No evaluation has been made on the climbing fibre varicosity distribution since at this age the VGlut2 marker is unspecific [43].

### Inhibition of Eph receptors in mature cerebellar slices also increases spine density in the proximal, but not in the distal domain of Purkinje cell dendrites

**Figure 2:**
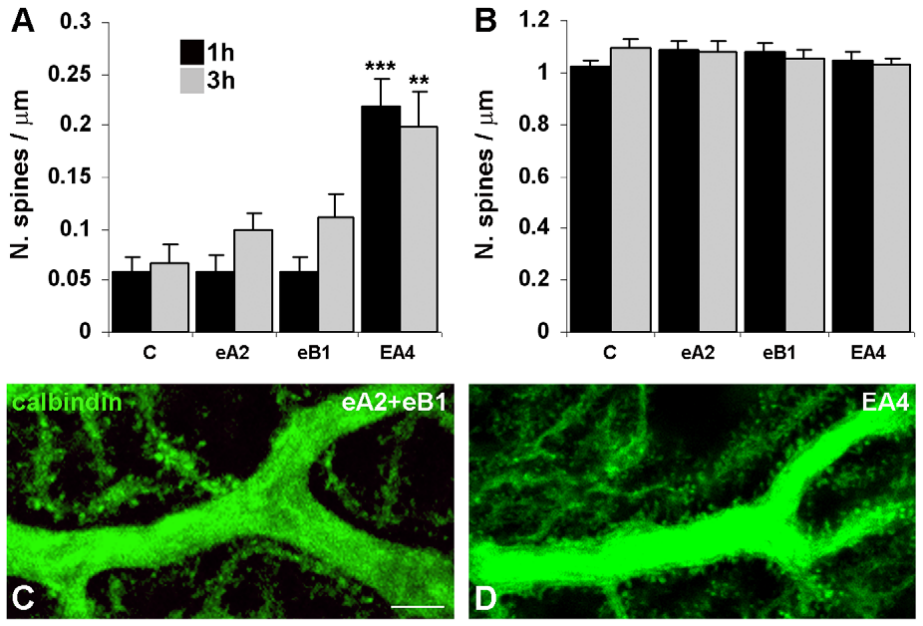
Purkinje cell morphological modifications in acute mature slices treated with ephrin ligand/and receptor/Fc chimeras. ***A, B,*** The histograms show the mean density of spines emerging from the proximal (***A***) and distal (***B***) dendritic domain after 1 and 3 hours of treatment. Only EphA4/Fc (EA4) treatment significantly increased spine density in the proximal compartment relative to the control treatment (C). **P<0.01, ***P<0.001, Student's t-test. Error bars indicate s.e.m.. eA2, ephrinA2/Fc; eB1, ephrinB1/Fc. ***C, D,*** Confocal images of calbindin-labelled proximal dendrites after 1 hour of treatment. The delivery of ephrinA2/Fc (***C***) or ephrinB1/Fc does not affect the morphology of the Purkinje cell proximal dendritic domain, which appears as smooth as in control conditions. On the contrary, the inactivation of EphA and EphB receptor pathways with EphA4/Fc increases spinogenesis in Purkinje cell proximal dendrites (***D***). Scale bar, 10 µm.

To examine whether Eph receptor activation/inhibition has similar effects on Purkinje cell spine densities in the developing and adult cerebellum, we also used acute mature cerebellar slices. Similar to what we observed in P10–11 organotypic slices, activation of class A and B Eph receptors did not cause significant changes in spine density along the proximal dendrites (Table 1B, Fig. 2A) (F_(2,57)_ = 0.005, *P*>0.99 at 1 hour and F_(2,81)_ = 0.99, *P*>0.37 at 3 hours by one-way ANOVA). Fig. 2C shows a representative example of the Purkinje cell proximal dendrite from an ephrinA2/Fc-treated slice. In contrast, inhibition of both classes of Eph receptors with EphA4/Fc significantly increased spine densities in proximal dendrites after both 1 hour (*P*<0.001, Student's t-test) and 3 hours (*P*<0.002, Student's t-test) (Table 1B, Fig. 2D). Quantitative evaluation of spine densities in the distal dendritic domain (Table 1B, Fig. 2B) revealed no differences between the experimental groups (F_(2,72)_ = 1.194, *P*>0.30 at 1 hour and F_(2,72)_ = 0.30, *P*>0.70 at 3 hours by one-way ANOVA for the treatment with ephrins/Fc; *P*>0.4 at 1 hour and *P*>0.15 at 3 hours by Student's t-test for the treatment with EphA4/Fc). These results, obtained with acute adult cerebellar slices, suggest an involvement of Eph receptor signalling in the maintenance of Purkinje cell spines selectively in the proximal dendritic domain.

### *In vivo* inhibition of Eph receptors in the adult cerebellum also triggers spinogenesis in the proximal, but not in the distal dendritic domain of Purkinje cells

**Figure 3:**
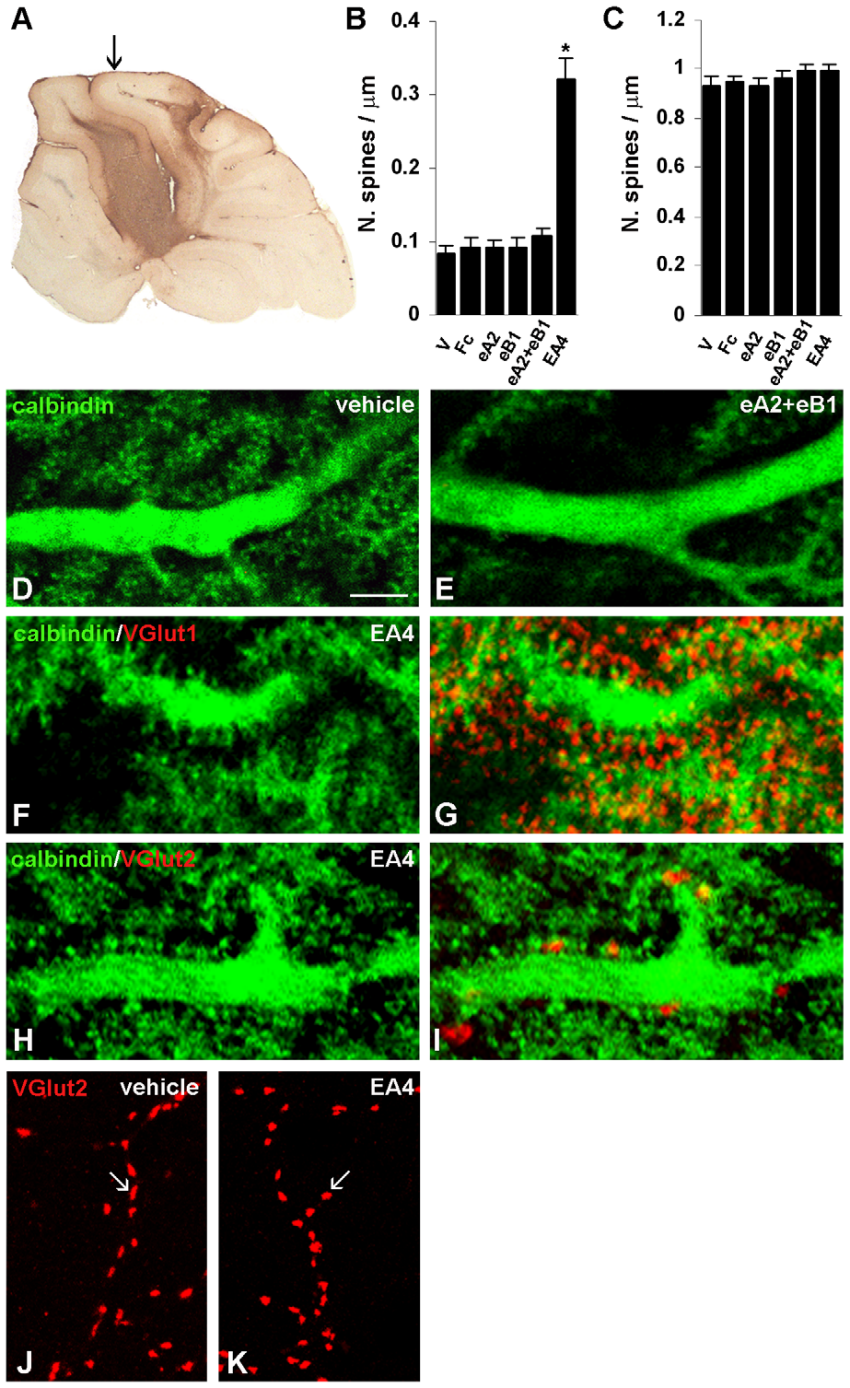
Purkinje cell morphological modifications after chronic infusion of ephrin ligand/and receptor/Fc chimeras in the cerebellum. ***A,*** The diffusion of the chimeras in the cerebellar parenchyma involves the two lobules adjacent to the insertion site of the minipump cannula (optical image). The arrow indicates the point in which the cannula penetrates into the cerebellum. ***B, C,*** The histograms show the mean density of spines emerging from the proximal (***B***) and distal (***C***) dendritic domain after 6 days of treatment. EphA4/Fc (EA4) treatment significantly increased spine density in the proximal compartment relative to the vehicle (V) and Fc controls (Fc). *P<0.05, Student-Newman-Keuls post-hoc method after one-way ANOVA. Error bars indicate s.e.m.. eA2, ephrinA2/Fc; eB1, ephrinB1/Fc. ***D, E, F, G, H, I,*** Confocal images of calbindin-labelled Purkinje cells (green) show proximal dendritic segments, which are typically smooth in the vehicle-treated cerebellum (***D***) and after ephrinA2/Fc plus ephrinB1/Fc administration (***E***). After treatment with EphA4/Fc, the proximal compartment appears fully covered by spines (***F, H***), which are contacted by parallel (***G***) and climbing fibres (***I***), as shown by VGlut1 and VGlut2 immunolabelling (red), respectively. ***J***, ***K***, Confocal images of VGlut2-labeled climbing fibre varicosities (red) in contact with the proximal compartment of Purkinje cells in vehicle- (***J***) and EphA4/Fc treated cerebella (***K***). Arrows indicate representative varicosities. Scale bar, 10 µm.

Because the climbing fibre input is altered in slices, we verified the results obtained in cerebellar slices by chronically infusing the Fc chimeras for 6 days with osmotic minipumps inserted into the cerebellum. Immunolabelling revealed a pronounced diffusion of the Fc proteins in the two lobules adjacent to the insertion site of the minipump cannula (Fig. 3A). Accordingly, we confined subsequent analyses to these cerebellar regions but excluding the tissue near the area damaged by the cannula. In addition to vehicle alone, we also infused Fc as a negative control. Treatment with ephrinA2/Fc, ephrinB1/Fc or both chimeras did not significantly alter spine densities (F_(4,511)_ = 0.76, *P*>0.50, one-way ANOVA) (Table 1C, Fig. 3D,E). In contrast, treatment with EphA4/Fc significantly increased spine densities in the Purkinje cell proximal dendritic compartment (F_(2,286)_ = 50.4, *P*<0.001, one-way ANOVA; *P*<0.05, post-hoc Student-Newman-Keuls method) (Table 1C, Fig. 3F,H). Consistent with the results obtained with slices, the new spines were contacted by parallel fibre varicosities (Fig. 3G).

Climbing fibre varicosities were detected on the proximal dendritic domains by labelling them with the antibody for the vesicular glutamate transporter 2 (VGlut2) (Fig. 3I). After 6 days of treatment there was no significant difference in the density of climbing fibre varicosities in EphA4/Fc treated cerebella as compared to vehicle-treated controls (*P*>0.49, Student's t-test) (Table 2A). However, EphA4/Fc treatment caused morphological modifications in the varicosities, including a significant reduction in the length of the major axis (*P*<0.001, Student's t-test), the ratio between major and minor axis (*P*<0.001, Student's t-test), the area (*P*<0.05, Student's t-test) and the perimeter (*P*<0.01, Student's t-test) (Table 2A). In contrast, we did not detect significant changes in the minor axis of the varicosities (*P*>0.05, Student's t-test). Overall, the EphA4/Fc treatment modified the shape of climbing fibre varicosities from elongated to rounded (Table 2A) (Fig. 3J and 3K).

**Table 2:** Morphological analysis of climbing fibre varicosities in different experimental approaches.

		Density	MA	ma	MA/ma	Area	Perimeter
		(n±s.e.m.)	(µm±s.e.m.)	(µm±s.e.m.)	(µm±s.e.m.)	(µm^2^±s.e.m.)	(µm±s.e.m.)
**A.**	**6 days of infusion**						
	Vehicle	0.322±0.020(n = 58)	1.410±0.054(n = 94)	0.550±0.024(n = 94)	2.930±0.165(n = 94)	0.841±0.048(n = 94)	3.998±0.111(n = 94)
	EphA4/Fc	0.303±0.018(n = 57)	1.090±0.054(n = 84)***	0.620±0.029(n = 84)	1.870±0.092(n = 84)***	0.699±0.053(n = 84)*	3.546±0.114(n = 84)**
**B.**	**EphB deficient mice**						
	EphB1−/−, EphB2+/+, EphB3−/−	0.423±0.029(n = 49)	1.610±0.081(n = 79)	0.610±0.032(n = 79)	3.100±0.229(n = 79)	1.034±0.074(n = 79)	4.498±0.175(n = 79)
	EphB1−/−, EphB2+/−, EphB3−/−	0.392±0.058(n = 45)	1.310±0.065(n = 80)**	0.610±0.038(n = 80)	2.360±0.115(n = 80)**	0.804±0.074(n = 80)*	3.854±0.158(n = 80)**

Mean values of climbing fibre varicosities density (expressed as number of climbing fibre varicosities per µm of dendritic length), major axis length (MA), minor axis length (ma), ratio (MA/ma), area and perimeter.

*P<0.05,

**P<0.01,

***P<0.001, Student's t-test.

Spine densities in the Purkinje cell distal dendritic domain of adult cerebella were not affected by any of the experimental conditions (F_(4,293)_ = 0.79, *P*>0.53 by one-way ANOVA for the treatment with ephrins/Fc; F_(2,175)_ = 1.43, *P*>0.24 by one-way ANOVA for the treatment with EphA4/Fc) (Table 1C, Fig. 3C). Therefore, the effects of Eph receptor inhibition in the intact mature cerebellum are fully in agreement with those observed *in vitro* in neonatal and adult cerebellar slices. Altogether, our findings implicate Eph receptor signalling in the control of spine density in the proximal dendritic domain of Purkinje cells, most likely by suppressing the intrinsic formation of new spines.

### *EphB* triple deficient mice exhibit increased spine density in the proximal dendrites of Purkinje cells

**Figure 4:**
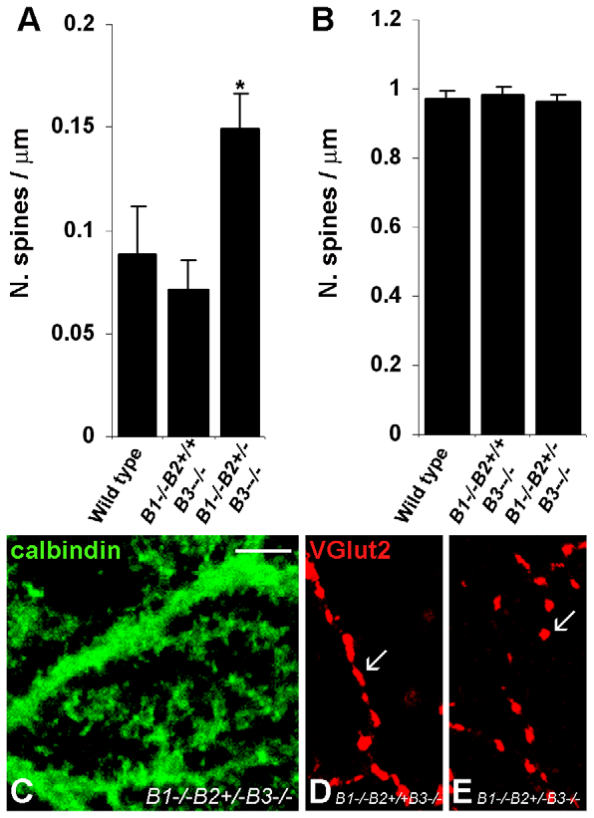
Purkinje cell morphology in double and triple EphB deficient mice. ***A, B,*** The histograms show the mean density of spines emerging from the proximal (***A***) and distal (***B***) dendritic domains in adult wild type, double *EphB1^−/−^*, *EphB3^−/−^* and triple *EphB1^−/−^*, *EphB2^+/−^*, *EphB3^−/−^* deficient mice. The spine density in the proximal compartment is significantly increased in triple mutant *EphB1^−/−^*, *EphB2^+/−^*, *EphB3^−/−^* versus wild type and double mutant *EphB1^−/−^*, *EphB3^−/−^* mice. *P<0.05, Student-Newman-Keuls post-hoc method after one-way ANOVA. Error bars indicate s.e.m. ***C,*** Confocal image of the calbindin immunolabelled cerebellar sagittal section from *EphB1^−/−^*, *EphB2^+/−^*, *EphB3^−/−^* mice shows a proximal compartment fully covered by spines. ***D***, ***E***, Confocal images of VGlut2-labeled climbing fibre varicosities contacting the proximal compartment of Purkinje cells in the cerebella of double (***D***) and triple (***E***) EphB-deficient mice. Arrows indicate representative varicosities. Scale bar, 10 µm.

To further confirm the suppressive effects of Eph receptor signalling on spinogenesis in the proximal domain of Purkinje cell dendrites, we used a genetic approach and analysed *EphB* knockout mice [7]. The mean spine density in the proximal dendrites of double *EphB1* and *EphB3* knockout mice was not significantly different from that in wild-type mice (Table 1D, Fig. 4A), whereas the spine density in the proximal dendrites of triple *EphB1*, *EphB2 EphB3*-deficient mice (*EphB1* ^−/−^, *EphB2* ^+/−^, *EphB3* ^−/−^) was significantly higher (Table 1D, Fig. 4A,C; F_(2,119)_ = 4.71, *P*<0.010, one-way ANOVA; *P*<0.05, post hoc Student-Newman-Keuls method).

The climbing fibre varicosities on the Purkinje cell proximal dendrites of triple *EphB*-deficient mice exhibited a significant reduction in the length of the major axis, the ratio between major and minor axis, the area and the perimeter as compared to double *EphB1*, *EphB3*-deficient mice (*P*<0.01, *P*<0.01, *P*<0.05, *P*<0.01 by Student's t-test, respectively; Table 2B; Fig. 4D,E). In contrast, the density and the length of the minor axis of varicosities were not significantly different between the two groups of *EphB*-deficient mice (*P*>0.49 and *P*>0.93 by Student's t-test, respectively; Table 2B). In addition, the spine density values in the distal domain of the Purkinje cell dendrites were not significantly different between wild-type and double *EphB1*, *EphB3*-deficient mice or triple *EphB1*, *EphB2*, *EphB3*-deficient mice (F_(2,145)_ = 0.11, *P*>0.89, one-way ANOVA) (Table 1D, Fig. 4B). These results suggest that class B Eph receptors are involved in the climbing fibre-mediated suppression of spinogenesis in the proximal dendritic domains of Purkinje cells.

### Eph receptor activation partially suppresses spinogenesis induced by inhibition of climbing fibre electrical activity in Purkinje cell proximal dendrites

**Figure 5:**
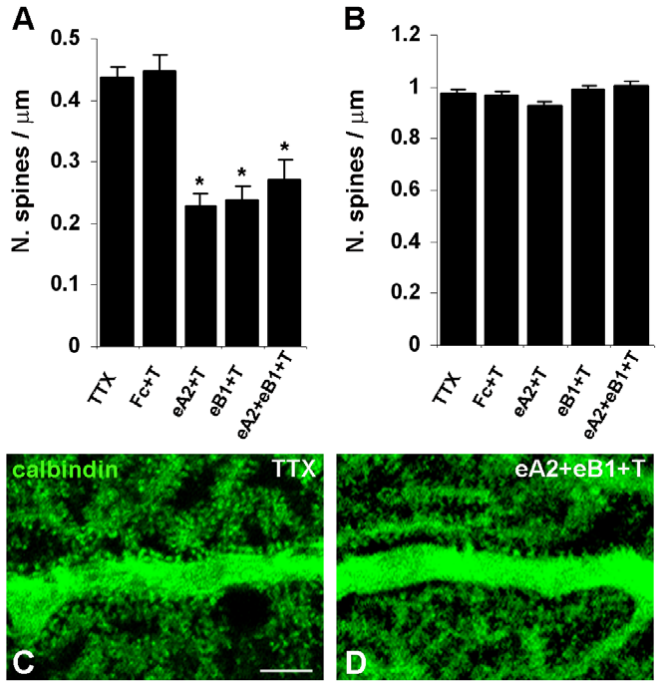
Purkinje cell morphological modifications after chronic infusion of tetrodotoxin, ephrin ligand/and receptor/Fc chimeras in the cerebellum. ***A, B,*** The histograms show the mean density of spines emerging from the proximal (***A***) and distal (***B***) dendritic domain after 6 days of treatment. The high density of spines induced by tetrodotoxin (with or without the concurrent infusion of control Fc) on the proximal compartment is significantly lowered by the simultaneous infusion of ephrin Fc chimeras. *P<0.05, Student-Newman-Keuls post-hoc method after one-way ANOVA. Error bars indicate s.e.m.. eA2, ephrinA2/Fc; eB1, ephrinB1/Fc; T, tetrodotoxin. ***C, D,*** Confocal images of the calbindin-labelled Purkinje cell proximal domain showing the changes in spine density induced by the treatment with tetrodotoxin (***C***) or tetrodotoxin plus ephrinA2/Fc and ephrinB1/Fc (***D***). Scale bar, 10 µm.

To determine whether Eph receptor signalling may be involved in climbing fibre-mediated suppression of spinogenesis, we examined the effects of Eph receptor activation on spinogenesis induced by pharmacological block of climbing fibre electrical activity. In a first group of mice, we administered the sodium channel blocker tetrodotoxin for 6 days through a minipump inserted in the cerebellar parenchyma in order to block the repressive action of climbing fibres on spinogenesis [24], [25]. Spine densities in proximal dendrites were significantly higher in tetrodotoxin-treated (Fig. 5C) than in vehicle-treated animals (*P*<0.001, Student's t-test; compare Table 1C and 1E). A similar increase was observed when Fc was added to tetrodotoxin (*P*<0.001, Student's t-test). In contrast, addition of ephrinA2/Fc, ephrinB1/Fc or both to tetrodotoxin (Fig. 5D) significantly reduced spine densities as compared to the tetrodotoxin or Fc plus tetrodotoxin controls (Fig. 5A, F_(4,372)_ = 19.4, *P*<0.001, one-way ANOVA; *P*<0.05, post hoc Student-Newman-Keuls method). However, Eph receptor stimulation did not completely abolish the effect of tetrodotoxin to completely restore the spine densities observed in vehicle-treated cerebella (F_(3,295)_ = 20.2, *P*<0.001, one-way ANOVA; *P*<0.05, post hoc Student-Newman-Keuls method; compare Table 1C and 1E). In contrast, the spine densities in the distal dendritic domains of Purkinje cells were not affected by treatment with tetrodotoxin alone or in combination with ephrin Fc chimeras (F_(4,357)_ = 2.31, *P*>0.05, one-way ANOVA) (Table 1E, Fig. 5B). These results demonstrate that Eph receptor activation by ephrins can partially restore spine pruning in the Purkinje cell proximal dendritic domains of tetrodotoxin-treated cerebella in the absence of climbing fibre activity, suggesting that ephrin/Eph signalling contributes to the spine inhibitory effect mediated by active climbing fibres.

## Discussion

Dendritic spine formation mainly occurs through two different mechanisms. Spines can be induced by presynaptic terminals or they can form by intrinsic mechanisms [28]. The latter pattern applies to the Purkinje cells. Numerous spines without presynaptic contact have been observed in these neurons under different conditions, particularly when granule cells degenerate before their parallel fibres have formed synapses or when adhesion between parallel fibres and Purkinje cells is compromised [39], [44]–[46]. When the climbing fibre input is deleted [20]–[23], the entire Purkinje cell dendritic territory is covered by spines that are innervated by parallel fibres. A similar hyperspiny transformation occurs when electrical activity is blocked by tetrodotoxin or ionotropic glutamate receptor antagonists [24], [25], [39]. Concomitantly, the climbing fibres undergo morphological modifications such as a reduction in the size of axonal varicosities and loss of synaptic contacts with Purkinje cells. The hyperspiny transformation induced by either lesion or block of activity is reversible. For example, olivary neurons surviving after lesion undergo collateral sprouting and reinnervate the denervated Purkinje cells. Morphological recovery of climbing fibres also occurs after removal of activity blockers. In both instances, the newly-formed spines in the proximal dendritic domain are lost [20], [21], [24]. These findings led to the hypotheses that: i) an activity-independent, intrinsic mechanism promotes spine growth over the whole Purkinje cell dendritic territory [19], [27] and ii) an activity-dependent spine-pruning action is exerted by the climbing fibres in the territory surrounding their synapses as a kind of lateral inhibition [19], [39]. The molecular mechanisms involved in this type of plasticity are poorly understood. This cerebellar plasticity brings to mind the intrinsic ability of denervated muscle fibres to express acetylcholine receptors along their entire length [47]. When the motoneuron makes contact with the muscle fibre, by means of its activity acetylcholine receptors cluster at the synapse and are depleted outside the synaptic area [48].

Several molecules can regulate spinogenesis by modulating actin filament dynamics in neurons [49]–[50]. Aside from glutamate receptors, scaffolding proteins of the postsynaptic density and adhesion molecules, Eph receptors and ephrins play an important role in dendritic spine formation and maintenance [3], [6], [8], [49]. The Eph receptors interact with the membrane-bound ephrins expressed by neighbouring cells, triggering bi-directional signals at sites of cell contact [3], [6], [8]. Multiple Eph receptors and ephrins are expressed in cerebellar neurons in different animal species, both during development and in adulthood [4], [30], [31], [33]–[37], [51], [52]. For example, several EphA receptors including EphA4 have been reported to be expressed in the dendrites of adult Purkinje cells [31], [53]–[55]. However, the role of Eph receptors and ephrins on spinogenesis in the cerebellum has not been addressed.

In this study, we have investigated whether Eph/ephrin signalling is involved in the regulation of dendritic spines in Purkinje cells of the cerebellum, and in particular whether it is involved in the repressive action of climbing fibre synapses on spinogenesis in nearby dendritic regions. We found that in both developing and mature cerebellar slices, application of ephrinA2/Fc and/or ephrinB1/Fc – which activate EphA and EphB receptors, respectively – has no effect on spine densities in both the proximal and distal dendritic domains of Purkinje cells. In contrast, we detected significant changes in spine density following the administration of EphA4/Fc, which inhibits the binding of endogenous ephrins to Eph receptors of both classes thus preventing receptor downstream signalling. Interestingly, in both developing and mature cerebellar slices EphA4/Fc induced intense spinogenesis in proximal Purkinje cell dendrites without affecting the distal domain. Furthermore, the hyperspiny transformation already occurred after one hour treatment of cerebellar slices with EphA4/Fc, and thus as rapidly as the ephrin- or Eph receptor-induced spine changes previously reported in hippocampal neurons [9], [10]. However, in contrast to the cerebellum, both Eph receptor activation and inhibition have been reported to affect dendritic spines in the hippocampus. EphB receptor activation in hippocampal neurons induces spine maturation [7], [9], [56], whereas block of both EphA and EphB receptors leads to a disorganized spine appearance and loss of mature spines [7], [9], [10].

The results obtained in slices were confirmed *in vivo* by chronic infusion of EphA4/Fc or ephrin Fc fusion proteins into the intact cerebellar parenchyma of adult mice. A higher spine density was detected in the Purkinje cell proximal dendritic domain after infusion of EphA4/Fc as compared to controls. In addition, the majority of the newly formed spines were contacted by parallel fibres, that we could identify by means of the immunostaining for VGlut1 that is selectively expressed in parallel fibres and not in climbing fibre terminals. These latter terminals, in fact, only express VGlut2 throughout the postnatal period and in adulthood (42). Therefore, in the presence of EphA4/Fc the parallel fibres invaded the proximal dendritic domain showing a competitive advantage over climbing fibres, as they do after chemical or surgical inhibition of climbing fibre activity [19].

Moreover, the climbing fibre inputs underwent morphological modifications, including a significant reduction in the dimensions of their varicosities. A similar reduction has also been observed following infusion of glutamate receptor antagonists due to a decrease in active synaptic contacts received by each climbing fibre varicosity and it has been attributed to a reciprocal competition between the two types of synapses [39].

Our *in vitro* and *in vivo* experiments suggest that inhibition of Eph receptor signalling by EphA4/Fc, which competes with endogenous Eph receptors for ephrin binding, induces intense spinogenesis in Purkinje cell proximal dendrites. However, EphA4/Fc may also activate reverse signalling through ephrins. We therefore analysed the cerebella of *EphB*-deficient mice (*EphB1* ^−/−^, *EphB2* ^+/−^, *EphB3* ^−/−^), in which not only EphB signalling, but also ephrin B reverse signalling is impaired. The high spine density we observed in proximal Purkinje cell dendrites in these mice suggests that Eph receptor signalling rather than ephrin reverse signalling suppresses spinogenesis. Immunostaining for VGlut2 further demonstrated morphological modifications in the climbing fibre varicosities of triple *EphB*-deficient mice that were similar to those induced by treatment with EphA4/Fc. Therefore, the pharmacological and genetic analyses together suggests that Eph receptor signalling may play a role in climbing fibre-mediated spine pruning in the proximal dendritic domain of Purkinje cells and in the accompanying changes in the morphology of climbing fibre varicosities [19], [57].

**Figure 6:**
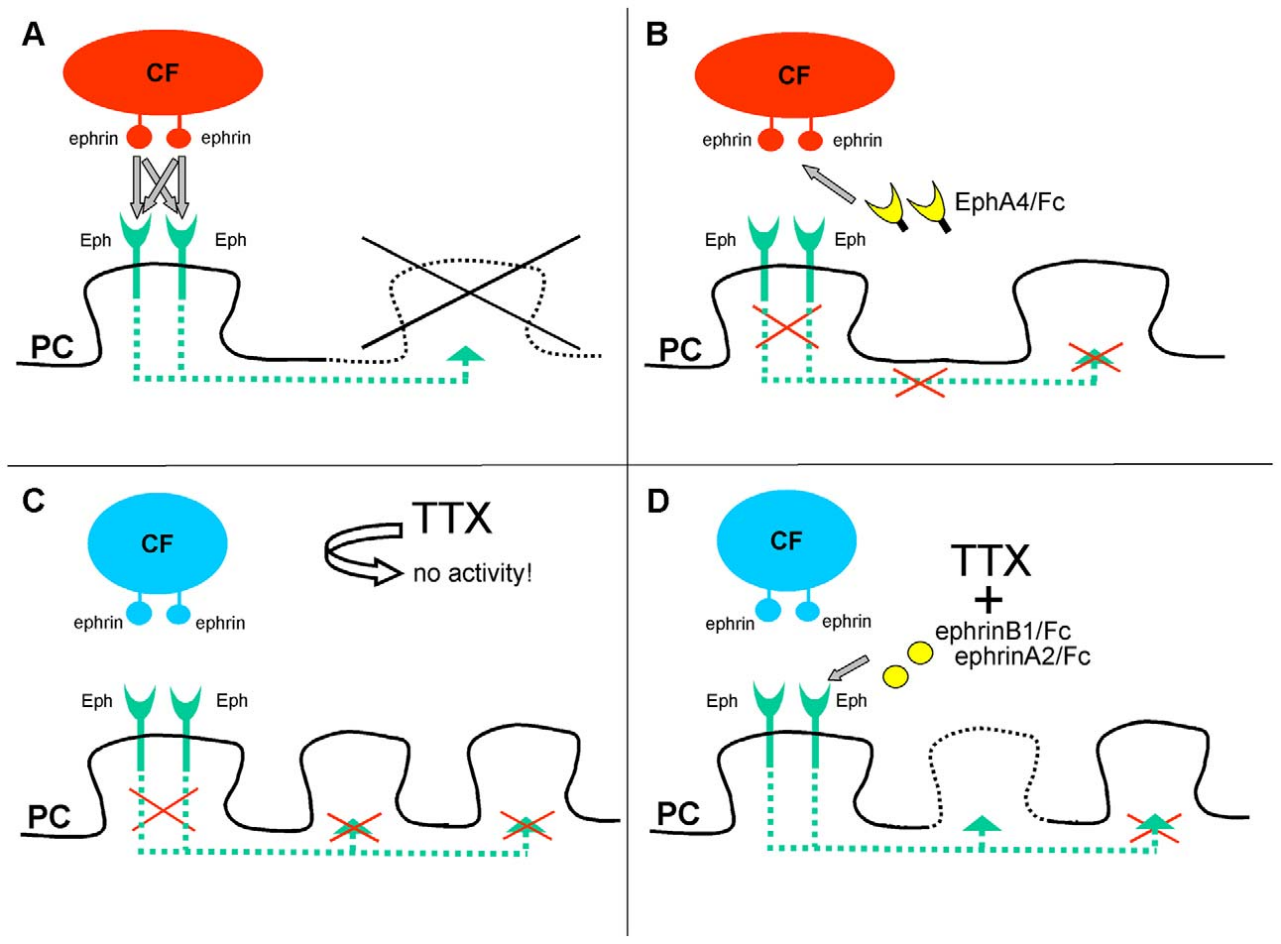
Model illustrating the mechanisms of ephrin pathways in the control of axonal competition in the cerebellar cortex. **A**, Under normal conditions, ephrins in presynaptic climbing fibre varicosity activate Eph receptor pathways in Purkinje cells to repress spinogenesis in the surrounding territory of the proximal dendrites. ***B***, Inhibition of the Eph receptor signalling releases the repression thus allowing spinogenesis. ***C***, Suppression of climbing fibre activity by tetrodotoxin (TTX) removes Eph receptor signalling and promotes spinogenesis. ***D***, Activation of Eph receptor signalling by ephrin chimeras in the absence of climbing fibre activity reduces the density of new spines. PC, Purkinje cell; CF, climbing fibre.

The spine changes observed in the cerebella of the *EphB*-deficient mice were different from those seen in the hippocampus of *EphB1* ^−/−^, *EphB2* ^−/−^, and *EphB3* ^−/−^ triple knockout mice and various combinations of *EphB* ^−/−^ double knockout mice, where decreased spine density and abnormal spine morphologies were observed [7]. These discrepancies are most likely attributable to the unique mechanisms underlying spine formation and pruning in the cerebellum. In particular, spines in the proximal dendritic compartment of Purkinje cells are under a tonic repressive action exerted by climbing fibres. The spine proliferation induced by the EphA4/Fc inhibitor or EphB gene inactivation implicate Eph receptor signalling in spine suppression (Fig. 6A,B).

To further confirm the involvement of Eph receptor signalling in climbing fibre-mediated suppression of Purkinje cell spinogenesis, we blocked electrical activity with tetrodotoxin, which leads to intense spine proliferation in the proximal dendritic domain accompanied by the loss of climbing fibre synaptic contacts with Purkinje cells (Fig. 6C) [19]. Importantly, the infusion of ephrin Fc chimeras to activate Eph receptors recapitulated the spine pruning effects induced by climbing fibre activity and partially reversed the effects of tetrodotoxin, thus limiting the intrinsic propensity of Purkinje cell to form spines on the proximal dendritic domain (Fig. 6D).

Multiple signal transduction pathways are activated by the binding of ephrins to Eph receptors, leading to actin rearrangements that can promote dendritic spine formation and maturation as well as spine retraction and loss [3], [8], [56], [58]–[60]. For example, the spine-promoting effects of EphB receptors in the hippocampus were shown to depend on increased activity of guanine nucleotide exchange factors such as Kalirin, Intersectin and Tiam1, which activate the Rho family GTPases Rac1 and Cdc42 to promote actin polymerisation and branching, as well as decreased activity of the RhoA exchange factor Ephexin5 [9], [56], [61], [62]. Furthermore, a pathway involving RhoA activation downstream of the focal adhesion kinase FAK was reported to maintain mature spine morphology downstream of EphB receptors [63]. In contrast, the RhoA exchange factor Ephexin1 has been implicated in EphA4-dependent spine retraction and loss [64]. Furthermore, the GTPase-activating protein α-chimerin can inhibit Rac1 downstream of EphA4 in neurons, which could suppress dendritic spines [65]–[68], and ß1 integrin inactivation was also implicated in spine retraction downstream of EphA4 [69]. Although the downstream targets of Eph receptors in the cerebellum have not yet been identified, regulation of Rho family GTPases may mediate Eph receptor-dependent inhibition of spinogenesis and/or spine pruning in the proximal dendritic domain of Purkinje cells. For example, recruitment of spine/synapse-promoting signalling molecules to active climbing fibre synapses may deplete them from the surrounding dendritic regions resulting in decreased spinogenesis and an overall loss of spines. Alternatively, Eph receptor-dependent signals that induce spine retraction and synapse loss [64] may be active in the dendritic regions surrounding active climbing fibre synapses, leading to loss of parallel fibre innervation. Regardless of the precise mechanism, our results uncover a new surprising facet in the regulation of dendritic spines by Eph receptor/ephrin signalling.

## Supporting Information

Figure S1 **Methodology of spine density evaluation on distal dendrites.** Images of Purkinje cell distal dendritic domain. Panels from A to E display a series of individual z-sections (Δ = 0.5 µm) of a dendritic segment. Spines were counted in the central section (panel C) of the series on each side of the segment as indicated by the two white dotted lines. Only spines emerging from the dendrite in this central section have been counted and are indicated by the white arrows. By looking at panel C it is possible to identify two more spine heads (red and yellow arrows) near the dendrite. These spines, although belonging to the same dendrite, as shown by their emergence sites in panels B and D respectively, have not been included in our density evaluation. Scale bar: 5.0 µm.
(TIF)
