# Supplementary figures and images for: Eph Receptors Are Involved in the Activity-Dependent Synaptic Wiring in the Mouse Cerebellar Cortex
Source: PLoS One. 2011 Apr 29;6(4):e19160. doi: 10.1371/journal.pone.0019160 (PMC3084771; doi:10.1371/journal.pone.0019160)

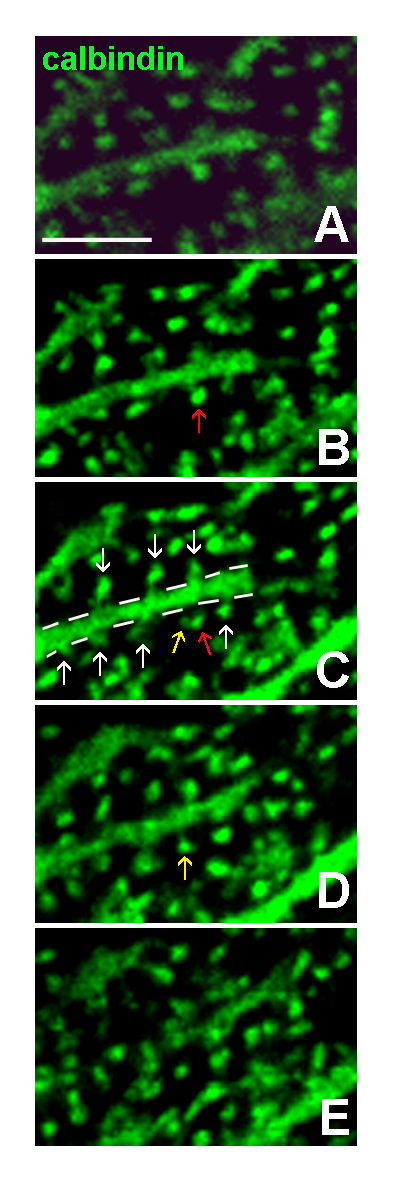

Supplement: Figure S1 — Methodology of spine density evaluation on distal dendrites. Images of Purkinje cell distal dendritic domain. Panels from A to E display a series of individual z-sections (Δ = 0.5 µm) of a dendritic segment. Spines were counted in the central section (panel C) of the series on each side of the segment as indicated by the two white dotted lines. Only spines emerging from the dendrite in this central section have been counted and are indicated by the white arrows. By looking at panel C it is possible to identify two more spine heads (red and yellow arrows) near the dendrite. These spines, although belonging to the same dendrite, as shown by their emergence sites in panels B and D respectively, have not been included in our density evaluation. Scale bar: 5.0 µm. (TIF) [file pone.0019160.s001.tif]
